# Remodeling Intestinal Flora with Sleeve Gastrectomy in Diabetic Rats

**DOI:** 10.1155/2014/196312

**Published:** 2014-08-04

**Authors:** Xiaofei Huang, Pan Weng, Huixin Zhang, Yingli Lu

**Affiliations:** Institute and Department of Endocrinology and Metabolism, Shanghai Ninth People's Hospital Affiliated to Shanghai Jiaotong University School of Medicine, No. 639, Zhizaoju Road, Shanghai 200011, China

## Abstract

*Objective*. As a complicated symbiotic system, intestinal flora is reported closely related to the development of type 2 diabetes recently. Sleeve gastrectomy is one of the approaches of bariatric surgery and could improve blood glucose control in type 2 diabetes patients. This study was to explore the relationship between remodeled intestinal flora and glucose metabolism in diabetic rats.* Methods*. 20 male diabetic rats were operated; 10 of them underwent sleeve gastrectomy, and 10 of them underwent sham operation. Meanwhile 10 male normal rats underwent sleeve gastrectomy as control. The animals' weight and FBG had been measured. The composition changes of intestinal flora were detected by 16S rDNA sequence analysis.* Results*. In diabetic rats, weight and fasting blood glucose decreased significantly after sleeve gastrectomy. However, there was no significant change for weight and blood glucose in normal rats after operation. The intestinal flora of diabetic rats reduced in the proportion of Firmicutes and increased in the proportion of Bacteroidetes after sleeve gastrectomy.* Conclusion*. The change of dominant microorganisms in intestinal flora might play an important role in the glucose metabolism.

## 1. Introduction

With the improvement of living standards, especially since the beginning of the 21st century, type 2 diabetes has become one of the global health problems. Type 2 diabetes also is the world's fourth largest cause of death, which has brought huge economic burden on countries in the world. According to the International Diabetes Federation (IDF) statistics, in 2011, the global diabetes has reached 366 million, and, by 2030, this figure is estimated to be 552 million [1].

At present, the main treatments for type 2 diabetes are balance nutrition, exercise, oral agents, and insulin therapy. However, there are still about 60% of diabetes patients who cannot reach the standards [2] for glycated hemoglobin. In addition, some oral hypoglycemic agents and insulin may result in weight gain, which may further impair blood glucose control. Thus, a new approach which can not only control blood glucose but also can lose body weight is urgently needed.

Bariatric surgery was originally developed solely as a weight loss therapy. In 1998, a retrospective study of 608 patients proved that bariatric surgery improves both obesity and type 2 diabetes patients with blood glucose control [3]. A recent research also indicated that bariatric surgery was far more effective than conventional medical therapy in the control of hyperglycemia in diabetes patients with severe obesity [4].

Surgical methods are the following four: vertical gastroplasty, adjustable gastric banding surgery, sleeve gastrectomy, and Roux-en-Y gastric bypass surgery. Using laparoscope, adjustable gastric banding surgery has become the standard bariatric surgery procedure in Europe and Australia. Roux-en-Y gastric bypass surgery shows the effect of both limiting the intake and slightly reducing the absorption, which leads to rapid and significant weight loss. However, there are several defects such as more complicated surgery procedures, more postoperative complications, or patients often needing long-term medication due to lack of nutrition.

Sleeve gastrectomy is one of surgical approaches that emerged in recent years. The operation is relatively simple, with laparoscopic, and has fewer complications. Despite the fact that there is not a long time since sleeve gastrectomy was applied, it has been found that sleeve gastrectomy takes similar effect with Roux-en-Y gastric bypass surgery in short term [5]. But the long-term efficacy requires further investigation.

However, the mechanisms of weight loss and improvement blood glucose control after bariatric surgery are not entirely clear yet. Reducing energy intake or absorption obviously could not fully explain. A large number of studies have reported gut hormones such as ghrelin, PYY, GLP-1 play an important role in losing weight and improving blood glucose after bariatric surgery [6–8]. Due to bariatric surgery involving the gastrointestinal tract, the interaction of the intestinal flora on metabolic diseases after bariatric surgery now becomes a new research area. More studies are about Roux-en-Y gastric bypass with obesity. However, the study of intestinal flora after sleeve gastrectomy in diabetes is still relatively rare [9, 10]. Compared with Roux-en-Y gastric bypass surgery, sleeve gastrectomy has less effects of intestinal structure, and it has not been reported whether it has a similar effect on the intestinal flora.

GK rat (Goto-Kakisaki rat) is a spontaneous diabetic animal model for gradual emergence of impaired insulin secretion and high blood glucose. This study uses sleeve gastrectomy on GK diabetes rats and normal control SD rats to observe the effect of surgery improved metabolism as well as the changes of the intestinal flora and explore the intestinal flora composition changes after the sleeve gastrectomy and its metabolic condition in different species rats.

## 2. Materials and Methods

20 male, 5-month-old, diabetic GK rats were randomly divided into sleeve gastrectomy group (SG) and sham-operation group (SO). Meanwhile, 10 male, 5-month-old normal blood glucose SD rats also received sleeve gastrectomy as control (SDSG). The animals were purchased from Shanghai Laboratory Animal Center, Chinese Academy of Sciences. They were kept in individual cages at room temperature (25°C), respecting light-dark cycles and receiving standard feed for two weeks before operation. The standard feed contained 352 kcal energy per gram, which was made up of 65.5% carbohydrates, 10.3% fat, and 24.2% protein. All feed was bought from Shanghai Slac Laboratory Animal Co. All rats' body weights were measured. The fast blood glucose (FBG) was measured using tail venous blood with Terumo glucometer. The rats' fecal samples were obtained one day before operation and kept in sterilized containers at −20°C.

Preoperative fasting of solids took place for 12 hours and 1 hour for liquid. Aseptic technique was used in the preparation of the operative field and the animal's abdominal wall, and antibiotic prophylaxis was applied with 100,000 IU penicillin per rat intramuscularly 30 minutes prior to the operation.

**Figure 1:**
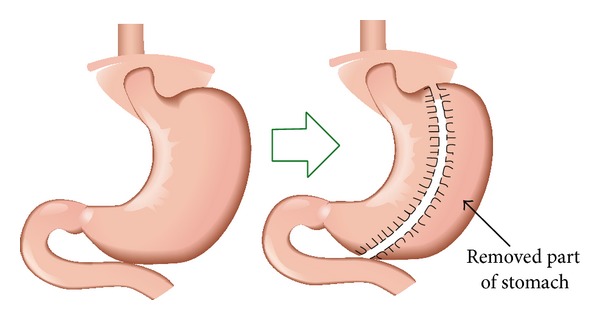
Schematic diagram of sleeve gastrectomy.

Intraperitoneal anesthetics were used with 10% chloral hydrate. Upper median laparotomy was conducted, the stomach was exposed, and its contents were emptied into the small bowel. In the SG group and SDSG group, adherences were freed and the vessels of the greater curvature were cauterized with a thermocautery, from the cardia all the way to the pylorus. A sleeve gastrectomy was conducted, resecting about 70% of the stomach [11] (Figure 1), while, in SO group, sham operations were performed. To avoid dehydration, 10 mL of saline solution was injected in the dorsal subcutaneous tissue immediately after surgery procedures. Upon resuming oral nutrition, all animals were given 5% glucose solution as a liquid diet for 1 day, after which solid nutrition was allowed gradually for 7 days (no more than 30 g/d). After the 8th day, the animals received water and standard feed. In addition, the rats were injected 10 mL of saline solution subcutaneously per day every morning for 3 days. All rats' daily food intake was measured. The body weight and FBG was measured at 1 month after operation. All experimental operations followed the Chinese animal experiment guideline.

Meanwhile, fecal samples were collected from each rat. DNA was extracted from fecal samples using the TIANGEN Fecal Genomic DNA extraction kit. Fecal samples were completely lysed with incubation at 70°C for 10 minutes in buffer GB and proteinase K. DNA from fecal samples was eluted in a final volume of 200 *μ*L elution buffer and stored at –20°C. The 16S rDNA was amplified with polymerase chain reaction (PCR) using broad-range bacterial primers (8F 5′-AGAGTTTGATCCTGGCTCAG-3′ 1391R 5′-GACGGGCGGTGTGTRCA-3′). PCR products were cloned and sequenced bidirectionally by Shanghai Biological Technology Co. And the sequence results were compared with BLAST and RDP database to determine the species of intestinal flora. The closest neighbors to our sequences among the BLAST and RDP databases were determined from local phylogenies using the maximum likelihood algorithm, and such sequences were used to represent species where possible. The proportions of specific bacteria were described in mean ± standard deviation (*x* ± SD).

Body weight, daily food intake and FBG data were expressed in mean ± standard deviation (*x*  ±  SD). All statistical analyses were conducted with the use of SAS software version 9.1 (SAS Institute). Analysis of variance was used to compare the proportions of specific bacteria, and Student's *t*-tests to compare numerical data.

## 3. Results

In SG group, 1 rat died during surgery and 3 rats died within 3 days after surgery. In SDSG group, 2 rats died within 3 days after surgery and 1 rat died 5 days after surgery. No rats died in SO group.

At the beginning of the study (0 week), the weight of the SG group and SO group rats had no significant difference (*P* = 0.928). One month after sleeve gastrectomy and sham operation, respectively, SG group and SDSG group rats' weights were both reduced, while SO group rats' body weight increased. Compared with SO group, SG group rats' weight had statistically significant difference (*P* = 0.042) (Table 1).

**Figure 2:**
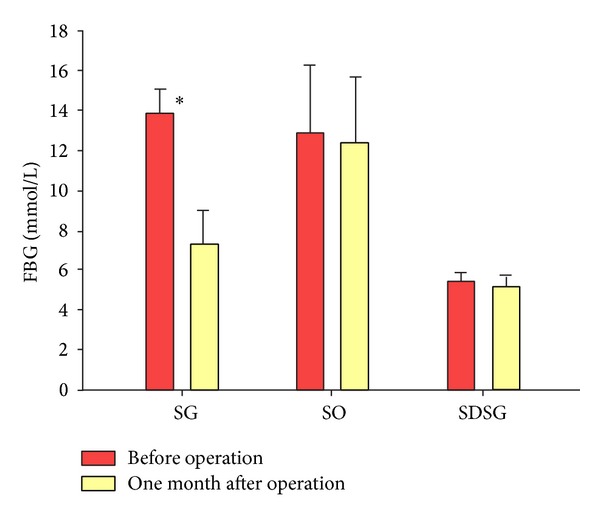
Changes of rat's FBG among groups. Sleeve gastrectomy group (SG), sham-operation group (SO), and sleeve gastrectomy of SD rats group (SDSG). Compared with SO group, *P < 0.05.

At the beginning of the study (0 week), the SG group and SO group rats' basis fasting blood glucose had no significant difference (*P* = 0.644) and was significantly higher than the SDSG group rats' glucose. One month after sleeve gastrectomy and sham operation, respectively, the fasting glucose levels of SO group and SDSG group rats also decreased, but the difference was not statistically significant (*P* > 0.05). Compared with SO group, SG group rats' fasting blood glucose had statistically significant difference (*P* < 0.05) (Figure 2).

**Figure 3:**
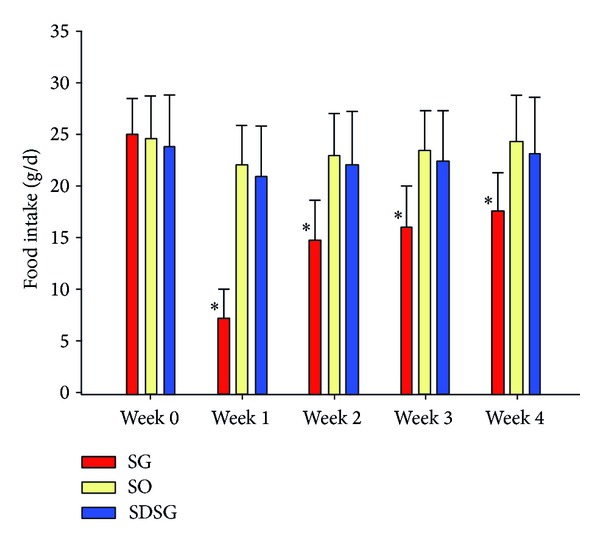
Changes of rat's food intake amount among sleeve gastrectomy group (SG), sham-operation group (SO), and sleeve gastrectomy of SD rats group (SDSG). Compared with SO group and SDSG group, *P < 0.05.

At the beginning of the study (0 week), the daily food intake had no significant difference among SG group, SO group, and SDSG group. At the first week after operation, each group had a decrease in food intake, especially the SG group. In the next three weeks, the food intake of all groups gradually increased. However, compared with SO group and SDSG group, the daily food intake of diabetic SG group rats had significant differences throughout the whole postoperation period (*P* < 0.05) (Figure 3).

**Figure 4:**
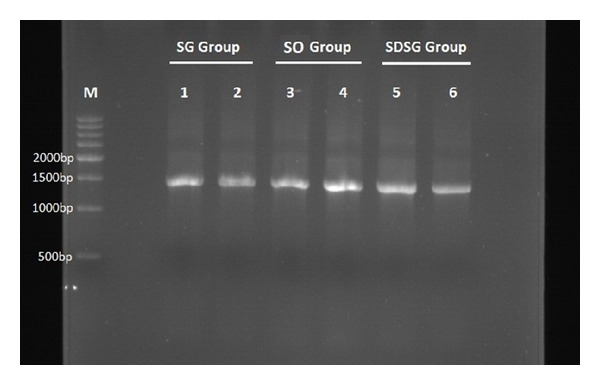
PCR amplification results of 16S rRNA gene 1 month after different operation procedures. Sleeve gastrectomy group (SG), sham-operation group (SO), and sleeve gastrectomy of SD rats group (SDSG).

**Figure 5:**
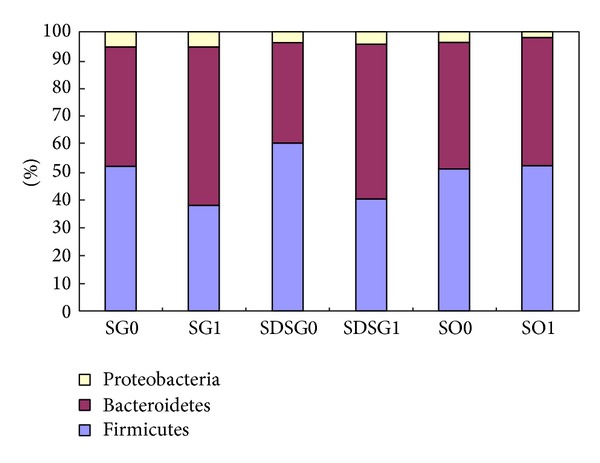
Composition of rat intestinal flora obtained by Sanger sequencing method before sleeve gastrectomy and one month after sleeve gastrectomy. Sleeve gastrectomy group (SG0, SG1), sleeve gastrectomy of SD rats group (SDSG0, SDSG1), and sham-operation group (SO0, SO1).

Intestinal flora 16S rDNA was amplificated by PCR and sequenced by Sanger sequencing method (Figure 4). Compared with BLAST and RDP database, the sequencing results indicated the intestinal flora was distributed in Firmicutes, Proteobacteria, and Bacteroidetes, three major phyla. In the two groups of GK rats before sleeve gastrectomy, the intestinal flora was roughly similar in composition. And the composition of intestinal flora of GK rats and SD rats was changed noticeably in the phylum level after sleeve gastrectomy. Firmicutes composition decreased significantly (52% ± 7.1% to 38% ± 6.4%; 60% ± 8.7% to 40% ± 7.5%, *P* = 0.031), while the proportion of Bacteroidetes increased significantly (43% ± 6.8% to 57% ± 8.9%; 36% ± 5.2% to 56% ± 8.3%, *P* = 0.044), and there was no significant change in the proportion of Proteobacteria before and after sleeve gastrectomy (5% ± 3.5% to 5 ± 5.2%; 4% ± 4.5% to 4% ± 6.4%, *P* > 0.05). There were no significant composition changes of intestinal flora in SO group before and after surgery (Figure 5).

**Figure 6:**
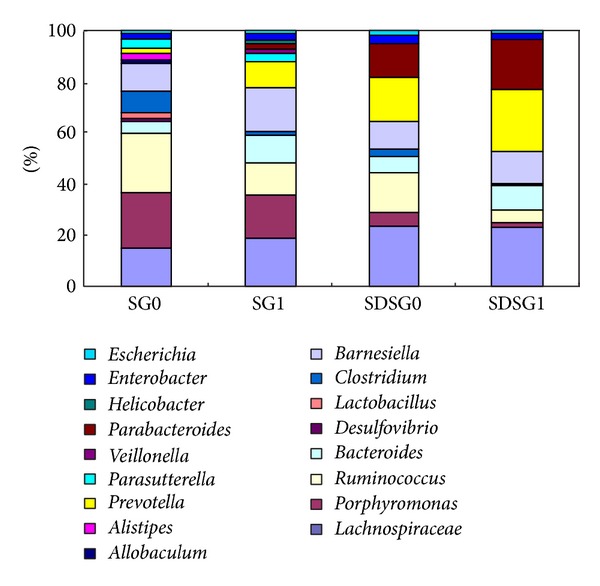
Taxonomic structure of diabetic rat intestinal flora before sleeve gastrectomy and one month after sleeve gastrectomy. Sleeve gastrectomy group (SG0, SG1) and sleeve gastrectomy of SD rats group (SDSG0, SDSG1).

In the level of genera, some kinds of Firmicutes,* Clostridium*, and* Ruminococcus* significantly decreased after surgery, while, in Bacteroidetes phylum, the proportion of* Prevotella* and* Parabacteroides* significantly increased (Figure 6).

## 4. Discussion

In this study, we first observed the impact of sleeve gastrectomy on metabolism of diabetic rats. Sleeve gastrectomy not only was able to reduce body weight, but also significantly improved glucose metabolism, where blood glucose tends to be normal after surgery. With these metabolic changes, we also observed that the intestinal flora both changed in normal SD rats and GK diabetic rats after sleeve gastrectomy, where the proportion of Firmicutes reduced and the proportion of Bacteroidetes rose. However, there was little effect on body weight and glucose metabolism in SD rats. We think the alteration of gut microbiota could reduce high glucose level, but it might not decrease normal glucose level to hypoglycemia.

Recently, many similar studies of bariatric surgery showed well blood glucose control in patients with type 2 diabetes, even when their body weight was normal before [12].

As we know, gastrointestinal hormone, involved in the mechanism of intestinal flora after bariatric surgery, is considered an important link to the degree of metabolic improvement. It is already reported that the proportion of Firmicutes and Gammaproteobacteria significantly increased after Roux-en-Y gastric bypass surgery in obese and nonobese individuals [13, 14]. The mechanism of causing these changes is unclear.

Changing the structure of the digestive tract, especially shorten the small intestine is speculated more conducive to facultative anaerobes than to strictly anaerobic bacteria such as Firmicutes. In addition, these changes make the incompletely digested food quickly enter the colon, which alters the dominant colon flora as the microenvironment changes [13]. As to sleeve gastrectomy, the length and structure of small intestine are not changed; our study also found no changes in the proportion of facultative anaerobes Proteobacteria. Therefore, there are other mechanisms playing a role in the change of other intestinal flora after sleeve gastrectomy.

In order to minimize the impact on the intestinal flora during surgery (antibiotics, postoperative liquid diet), we collected samples one month after surgery. Previous studies have shown that human gut flora is influenced by antibiotics, but just in the last 30 days [15]. In addition, the weight, blood glucose, and other major changes of metabolic parameters are the lowest 2–4 weeks after sleeve gastrectomy in our previous studies. It means this is the moment that the degree of improving effect of metabolism is the strongest. Consider these two above factors; we chose one month after sleeve gastrectomy as the time to research the relation between the intestinal flora and metabolism.

Ley et al. found Firmicutes and obesity are closely linked. He recognized that Firmicutes have the ability to produce gas of H_2_; these bacteria will ferment plant polysaccharide to produce SCFA and H_2_ gas at the same time, where SCFA provides additional energy for host, and it indeed found that the higher proportion of Firmicutes is in obese individuals [16]. In our study, there was also higher proportion of Firmicutes in obese SD rats than diabetic GK rats, but without statistically significant difference. We also found that, along with weight loss, the proportion of Firmicutes significantly decreased after sleeve gastrectomy. We think both of the reduction of food intake and intestinal bacteria result in weight loss in rats after sleeve gastrectomy.

Up to now, the study of intestinal flora is mostly focused on observation. It still could not select certain types of intestinal flora to be researched. Therefore, the flora changes found in this study may be the reason for metabolism condition being improved. However, the change of gut microbiota might be the result from improvements of metabolism. The relationship between the intestinal flora and metabolism remains to be further explored.

## Figures and Tables

**Table 1:** Changes of rat's body weight among groups. Sleeve gastrectomy group (SG), sham-operation group (SO), and sleeve gastrectomy of SD rats group (SDSG).

Time	Weight (g)			
	SG group (*n* = 10)	SO group (*n* = 10)	*P*	SDSG group (*n* = 10)
Before operation	362.8 ± 9.3 (*n* = 10)	361.5 ± 25.0 (*n* = 10)	0.928	412.0 ± 33.6 (*n* = 10)
One month after operation	332.2 ± 34.6 (*n* = 6)	379.2 ± 11.6 (*n* = 10)	**0.042**	397.5 ± 53.9 (*n* = 7)
*P*	0.139	0.245		0.502
